# HIV-Induced Apoptosis: Host Defense and Viral Strategy

**DOI:** 10.3390/biology14121680

**Published:** 2025-11-26

**Authors:** David Chisompola, Phinnoty Mwansa, John Nzobokela, Magdalene Ameka, Annet Kirabo, Antentor Hinton, Sepiso K. Masenga

**Affiliations:** 1 HAND Research Group, School of Medicine and Health Sciences, Mulungushi University, Livingstone 10101, Zambia; d.chisompola@gmail.com (D.C.); phinnotydariomwansa@gmail.com (P.M.); 2 Department of Pathology, Ndola Teaching Hospital, Ndola 10101, Zambia; johnnzojoes@gmail.com; 3 KAVI Institute of Clinical Research, University of Nairobi, Nairobi 19676-00202, Kenya; magdaleneameka@gmail.com; 4 Department of Medicine, Vanderbilt University Medical Center, Nashville, TN 37203, USA; annet.kirabo@vumc.org; 5 Vanderbilt Center for Immunobiology, Vanderbilt Institute for Infection, Immunology and Inflammation, Vanderbilt University Medical Center, Nashville, TN 37203, USA; 6 Department of Molecular Physiology and Biophysics, Vanderbilt University Medical Center, Nashville, TN 37203, USA; antentor.o.hinton.jr@vanderbilt.edu; 7 Vanderbilt Institute for Global Health, Vanderbilt University Medical Center, Nashville, TN 37203, USA; 8 Department of Cardiovascular Science and Metabolic Diseases, Livingstone Center for Prevention and Translational Science, Livingstone 10101, Zambia

**Keywords:** HIV, apoptosis, pyroptosis, inflammasome, Bcl-2, gp120, immune activation, viral persistence

## Abstract

HIV-induced apoptosis is a contradictory complicated phenomenon that occurs at the intersection of viral persistence and host defense. HIV primarily affects CD4 T cells during an infection, causing widespread immune cell death through both direct infection and indirect (bystander) mechanisms. This immunopathologic process is caused by viral proteins such as Tat, Nef, Env, and Vpr, which modify host signaling cascades such as the PI3K/Akt, p53, NF-κB, and mitochondrial pathways. Dysregulation of pro- and anti-apoptotic mediators, particularly Bax, Bcl-2, and caspase activation, which results in mitochondrial depolarization, oxidative stress, and cytochrome c release, exacerbates immune depletion. Although apoptosis serves as a host antiviral mechanism to limit viral replication and spread, HIV exploits it to evade immune surveillance and establish chronic infection. HIV pathogenesis, which includes lymphoid tissue destruction, microbial translocation, and persistent inflammation, is significantly influenced by apoptosis of both infected and bystander cells. Furthermore, alterations in death receptor signaling (Fas/FasL and TNF pathways) and mitochondrial dysfunction highlight the delicate balance between immune defense and viral manipulation. Despite considerable progress in antiretroviral therapy, immune restoration is still incomplete due to ongoing apoptotic loss and immune exhaustion. This review examines the biological mechanisms underlying HIV-induced apoptosis, evaluates the dual role of cell death in host defense versus viral persistence, and highlights novel therapeutic targets intended to restore immune homeostasis and reduce HIV-associated immunopathology.

## Simple Summary

When the Human Immunodeficiency Virus (HIV) infects a person, it triggers a process called apoptosis, a form of programmed cell death that is normally used by the body to eliminate damaged or infected cells. This review explores the double-edged role of this process in HIV infection. While apoptosis is initially a defense mechanism to limit the virus, HIV cleverly hijacks it to kill a massive number of uninfected immune cells, leading to a severe weakening of the immune system. This widespread cell death is a major reason why patients continue to have health problems even with effective antiviral medication. Our paper explains the complex mechanisms behind this phenomenon and evaluates new treatment strategies aimed at blocking this harmful cell death. By protecting the immune system from this collateral damage, these new approaches could improve the quality of life for people living with HIV and are a vital step toward a functional cure.

## 1. Introduction

More than four decades into the HIV pandemic, the virus still eludes immune responses and global health initiatives [1]. 40.8 million people still live with HIV despite tremendous advancements in biomedicine, and 1.3 million new cases were reported in 2024 alone. This implies that the epidemic is still ongoing [2]. HIV endures due to molecular strategies, particularly its capacity to take over immune processes and transform them into mechanisms that hasten immune destruction, in addition to social injustices and care gaps [3]. HIV selectively targets CD4^+^ T cells, causing a series of immune activation, exhaustion, and apoptosis that disrupts the immune system from within [3,4].

Antiretroviral therapy (ART) has revolutionized HIV-related morbidity and mortality by suppressing viral replication, but a persistent problem persists. The problem is that many patients receiving long-term treatment continue to lose CD4^+^ T cells, and their immune competence is not fully recovered [5,6]. As the disease progresses from clinical latency to acquired immunodeficiency syndrome (AIDS), CD4^+^ T cells depletion continues to be the primary indicator of HIV infection [7]. The interaction between immune activation, viral replication, and programmed cell death is highlighted by this slow immune system destruction [7].

Apoptosis has been identified as a key process among the different mechanisms proposed to explain CD4^+^ T-cell loss [8]. The large population of uninfected bystander cells exposed to viral proteins, inflammatory cytokines, or immune dysregulation is also impacted, in addition to effectively infected cells [8,9]. Despite successful ART, this pervasive programmed cell death weakens the immune system, encourages chronic inflammation, and helps viral reservoirs persist [10,11].

However, this phenomenon is defined by a fundamental paradox. One of the most fascinating aspects of HIV pathogenesis is the coexistence of opposing roles that continue to influence therapeutic and cure-oriented studies. Is HIV-induced apoptosis a viral adaptation that uses the host self-destruct mechanism to disable immunity and ensure its own survival, or is it an antiviral host defense strategy intended to limit infection by eliminating infected cells? In this review, we highlight the molecular mechanisms underlying HIV-induced apoptosis, critically analyze its dual role in host defense and viral persistence, and highlight new therapeutic approaches targeted at reducing HIV-associated immunopathology and restoring immune homeostasis.

## 2. Mechanisms of HIV-Induced Apoptosis

A cell goes through a strictly controlled process of self-destruction called apoptosis, as a host defense mechanism to remove abnormal, infected, or superfluous cells [12]. Evidence suggests that in HIV infection, uninfected bystander cells die through pyroptosis (an inflammatory type of programmed cell death), in addition to infected cells undergoing apoptosis [9]. It is interesting to note that only about 5% of cell death during HIV infection happens in directly infected cells, with the remaining 95% happening in nearby uninfected cells [13], underscoring the virus’s wider harm.

Numerous direct and indirect pathways can cause apoptosis to be activated in HIV infection. HIV proteins such as Tat interfere with normal cellular function, whereas viral envelope glycoproteins directly interact with host receptors such as the CD4 and CXCR4. Moreover, the cells’ self-destruct pathways are further triggered by cellular damage caused by mechanisms such as viral DNA integration and the activity of HIV protease and integrase enzymes. Apoptotic signaling is indirectly increased during infection by persistent immune activation, and targeted destruction by immune cells, especially CD8^+^ T cells, also contributes to apoptotic signaling, amplifying cell loss during infection [14]. The self-destruct cascade is triggered by actions of other immune cells, such as natural killer cells, B cells, and various surface markers.

### 2.1. Extrinsic Pathway Activation: Death Receptor Signaling

When particular cell surface death receptors (DRs) in the tumor necrosis factor (TNF) receptor superfamily are activated by their corresponding ligands, the extrinsic pathway is triggered [15,16]. This signaling mechanism ensures that damaged, infected, or superfluous cells are eliminated through programmed cell death, which is a crucial immune regulatory process. Among the most well-characterized death receptor systems are the Fas/FasL and TNF/TNF Receptor 1 (TNFR1) pathways, both of which converge on the activation of initiator caspases that execute apoptosis [15,17,18]. In the context of HIV infection, three key death receptor-mediated sequences of events occur, such as the Fas/FasL, TNF-related apoptosis-inducing ligand (TRAIL), and tumor necrosis factor-alpha (TNF-α), which have been implicated in the induction of CD4^+^ T-cell apoptosis and immune system dysregulation.

#### 2.1.1. Fas/FasL Signaling Cascade

The Fas/FasL signaling chain of events plays a vital role in mediating cytotoxic apoptosis, which is essential for maintaining immune homeostasis and eliminating cells infected by pathogens [19,20]. During an immune response, activated effector cells, particularly CD8^+^ cytotoxic T lymphocytes (CTLs) and natural killer (NK) cells, upregulate the expression of FasL on their surfaces [21]. FasL can exist in either a membrane-bound or soluble form, both of which interact with the Fas receptor (CD95/APO-1) expressed on target cells. Upon ligand interaction, Fas receptors trimerize, initiating the recruitment of the adaptor protein Fas-associated death domain (FADD) through homophilic death domain (DD) interactions [20,22].

FADD subsequently recruits procaspase-8 via death effector domain (DED) interactions, forming the death-inducing signaling complex (disk) [23,24,25]. Procaspase-8 molecules undergo proximity-induced activation within the disk, which results in their cleavage into active caspase-8. Depending on the type of cell, the downstream signaling varies. Apoptosis is carried out quickly and effectively in Type I cells, when caspase-8 directly activates effector caspases-3 and -7 [25,26]. The pro-apoptotic BH3-only protein Bid is cleaved into its truncated form (tBid) by caspase-8 in the mitochondrial (intrinsic) amplification pathway, which is used the Type II cells [27,28]. By promoting mitochondrial outer membrane permeabilization (MOMP), cytochrome c release, and caspase-9 activation through the apoptosome complex, tBid translocates to the mitochondria and amplifies the apoptotic signal [18,29].

The Fas/FasL cascade helps in immune regulation and contraction in addition to its function in removing pathogen-infected or transformed cells [22]. Fas/FasL signaling allows activated T cells to undergo activation-induced cell death (AICD) after antigen clearance, preventing excessive immune activation and maintaining peripheral tolerance [30,31]. But these processes can also cause bystander apoptosis, in which nearby uninfected Fas-expressing cells undergo apoptosis due to activated T cells expressing FasL, resulting in tissue damage and immune dysregulation [32].

#### 2.1.2. TNF/TNFR1 Signaling Cascade

TNF receptor 1 (TNFR1) and TNF receptor 2 (TNFR2), two structurally similar but functionally different receptors, are how tumor necrosis factor (TNF) exerts its biological effects. TNF can affect a variety of physiological and pathological processes because TNFR1 is widely expressed in practically all nucleated cell types [33]. TNFR2 expression, on the other hand, is more limited and is mainly present on immune cells such as regulatory T cells and endothelial cells, where it influences tissue repair and immune regulation [34,35]. Immune cells produce TNF in response to danger-associated molecular patterns (DAMPs) and pathogen-associated molecular patterns (PAMPs) [15,36].

By triggering the transcription of pro-inflammatory cytokines, chemokines, and adhesion molecules in target cells, TNF functions as a master regulator of inflammation [37,38,39]. Ubiquitination-dependent recruitment of adaptor proteins to TNFR1-associated signaling complexes initiates both NF-κB and MAPK signaling cascades, which are the primary mediators of inflammatory gene activation [40]. Significantly, depending on the cellular context, the presence of regulatory proteins and post-translational modifications of important signaling molecules signaling through TNFR1 determines cell fate outcomes such as cell survival, apoptosis, or necroptosis [18]. In order to preserve immunological homeostasis and regulate inflammatory reactions, pro-survival and pro-death cues are integrated by TNF/TNFR1 signaling, which serves as a crucial decision point [41].

The cytoplasmic death domain (DD) of TNFR1 is exposed when the trimeric TNF cytokine binds to three TNFR1 molecules through ligand interaction, causing receptor trimerization and conformational changes [42]. The membrane-associated Complex I and the cytosolic Complex II, two successive signaling complexes with different roles, are assembled as a result of this event, which also starts the recruitment of adaptor proteins [41,43].

In Complex I at the plasma membrane, TNFR1-associated death domain protein (TRADD) serves as a scaffold that recruits RIPK1, TRAF2, and cellular inhibitors of apoptosis proteins E3 ligases (cIAP1 and cIAP2) [44,45,46]. These adaptor molecules promote K63-linked ubiquitination of RIPK1, creating a docking platform for the recruitment of the linear ubiquitin chain assembly complex (LUBAC) [47]. Therefore, LUBAC further stabilizes Complex I and facilitates the recruitment and activation of downstream signaling molecules. These include the transforming growth factor-beta (TGF-β)-activated kinase 1 (TAK1) complex, composed of TAK1 and its adaptor proteins TAB2 and TAB3, and the inhibitor of κB (IκB) kinase (IKK) complex, which consists of the NF-κB essential modulator (IKKγ, also known as NEMO), IKKα, and IKKβ [48,49]. Activation of these complexes triggers the IKK and mitogen-activated protein kinase (MAPK) cascades, leading to the transcriptional activation of NF-κB–dependent inflammatory genes. Furthermore, the activation of NF-κB results in the transcription of pro-survival and pro-inflammatory genes, such as Bcl-2, cFLIP, and various cytokines and chemokines [50]. This pro-survival arm ensures that TNF signaling primarily supports inflammation resolution and tissue homeostasis under physiological conditions.

When the integrity of Complex I is disrupted, such as during cellular stress, HIV-1 infection, oxidative damage, or when transcriptional responses are impaired, RIPK1 undergoes deubiquitination and dissociates from the membrane complex [51,52,53]. It then interacts with cytosolic proteins to form a secondary signaling platform known as Complex II or the ripoptosome. This cytosolic complex contains TRADD, FADD, RIPK1 (often in a cleaved or inactive state), and procaspase-8 [54]. Functionally analogous to the disk, Complex II promotes the activation of caspase-8, which subsequently cleaves and activates downstream effector caspases (caspase-3, -6, and -7) as well as the BH3-only protein Bid [27,55], linking the extrinsic apoptotic pathway to mitochondrial-mediated apoptosis.

In cells where caspase-8 activity is inhibited or absent, RIPK1 can interact with RIPK3 and mixed lineage kinase domain-like pseudokinase (MLKL) to form the necrosome, triggering necroptosis, a regulated form of necrotic cell death [56,57]. In order to prevent the spread of intracellular pathogens and preserve tissue integrity, the apoptotic arm of TNF signaling, in particular, acts as a vital fail-safe mechanism to eradicate irreversibly damaged or infected cells.

#### 2.1.3. Immune Cell-Mediated Cytotoxicity via Death Receptors

The cytotoxic response triggered by CD8^+^ cytotoxic T lymphocytes (CTLs) is a significant illustration of extrinsic apoptotic pathway activation in host defense [58]. Through granule exocytosis and death receptor engagement, these specialized effector cells are essential in the removal of cancerous and HIV-infected cells. When foreign antigenic peptides presented by major histocompatibility complex (MHC) class I molecules on the surface of target cells are recognized by T-cell receptors (TCRs), CTLs polarize their cytotoxic machinery toward the target, creating a specialized contact site known as the immunological synapse [59]. This structure ensures the precise delivery of cytotoxic molecules by facilitating directed secretion and signal transduction between the effector and target cell. The extrinsic apoptotic signaling cascade is initiated by this interaction, which encourages the upregulation and surface expression of FasL. FasL initiates receptor trimerization and the recruitment of FADD adaptor proteins by binding to its cognate receptor Fas (CD95) on the target cell. Procaspase-8 is then recruited and activated by FADD to form the disk [58]. After cleaving and activating downstream effector caspases, especially caspase-3, activated caspase-8 causes the controlled disintegration of cellular structures and ultimately apoptosis.

In parallel, CTLs use the perforin/granzyme pathway as a complementary cytotoxic mechanism [60,61]. Perforin, stored within cytotoxic granules, forms transient pores in the target cell membrane, allowing entry of granzyme B, a potent serine protease [12,62,63]. Once inside, granzyme B either cleaves Bid, a pro-apoptotic BH3-only member of the Bcl-2 family, or directly activates caspase-3, the apoptosis executioner. The extrinsic and intrinsic apoptotic pathways are linked when tBid translocates to mitochondria, causing cytochrome c release, mitochondrial outer membrane permeabilization (MOMP), and caspase-9 activation [64,65]. Additionally, granzyme B can activate procaspase-8, reinforcing apoptotic signaling and ensuring cell death even in the presence of inhibitory mechanisms [64,66].

However, the Fas/FasL and perforin/granzyme pathways constitute a dual and synergistic cytotoxic strategy, enabling CTLs to effectively and irreversibly eliminate infected or transformed cells. This coordinated mechanism is essential not only for controlling pathogen replication and tumor growth but also for maintaining immune homeostasis by removing aberrant or damaged cells without provoking excessive inflammation.

### 2.2. Direct Mechanisms: Viral Proteins and Intracellular Signaling

One of the main factors influencing viral pathogenicity is the HIV envelope (Env) glycoprotein gp120 subunit, which mediates both viral entry and direct cytopathic effects on host immune cells [67,68]. In addition to facilitating viral entry, its interaction with the CD4 receptor and co-receptors CXCR4 or CCR5 can aberrantly activate apoptotic signaling in uninfected bystander CD4^+^ T cells, contributing significantly to immunopathogenesis [4,69,70,71].

CXCR4-mediated signaling can induce mitochondrial depolarization and cytochrome c release, further amplifying caspase-dependent apoptotic cascades. Env gp120 subunit interactions also disturb intracellular calcium homeostasis and activate the p38 MAPK signaling pathway, both of which contribute to mitochondrial dysfunction and DNA fragmentation [72,73]. Nevertheless, Env gp120 subunit binding to CCR5 also modifies Bcl-2 family proteins and activates pro-apoptotic transcription factors such as p53, changing the intracellular balance in favor of apoptosis [69]. Env gp120 subunit–CD4/CXCR4/CCR5 interactions are a potent mechanism of HIV-mediated cytotoxicity, which causes progressive CD4^+^ T-cell depletion, a key feature of HIV immunopathogenesis, and widespread bystander apoptosis.

HIV accessory proteins, such as Tat, Nef, and Vpr, are crucial in inducing apoptosis via oxidative and mitochondrial pathways [74,75]. By attaching to the TAR region of developing viral RNA, the Tat protein (trans-activator of transcription) not only increases the expression of viral genes but also causes harm to uninfected cells [76]. Extracellular Tat has the ability to enter nearby cells and interfere with mitochondrial function by promoting reactive oxygen species (ROS) production and impairing mitochondrial membrane potential. These changes sensitize cells to apoptosis by activating stress kinases such as JNK and p38 MAPK, as well as promoting cytochrome c release [77].

Similarly, Nef (negative factor) modifies intracellular signaling pathways to promote immunological dysregulation and apoptosis. It increases the expression of FasL on infected cells, decreases the expression of anti-apoptotic Bcl-2, and interferes with the PI3K/Akt survival pathway [10,78]. Oxidative stress brought on by Nef further compromises mitochondrial integrity, making both infected and uninfected immune cells more vulnerable to apoptosis.

The Vpr protein (viral protein R) exerts a pro-apoptotic role by causing cell cycle arrest at the G2/M phase, a state that favors viral replication but compromises host cell survival [79,80]. By interacting with the mitochondrial permeability transition pore complex, Vpr promotes cytochrome c release and loss of mitochondrial membrane potential, which triggers caspase-9–mediated apoptosis. It also increases ROS generation and triggers the ATR–Chk1 DNA damage response pathway, resulting in further cell death [81]. Tat, Nef, and Vpr work in concert to increase the apoptotic burden in HIV infection by causing oxidative stress, mitochondrial dysfunction, and cell cycle dysregulation.

However, activation of the mitochondrial (intrinsic) apoptotic pathway is a significant downstream effect of HIV-induced signaling [82]. The Bcl-2 family of proteins tightly controls this balancing of anti-apoptotic members Bcl-2 and Bcl-xL against pro-apoptotic members Bax, Bak, and Bid [83]. Viral proteins and aberrant signaling cause pro-apoptotic factors to be upregulated and anti-apoptotic defenses to be suppressed in HIV-infected and bystander cells, tipping the balance toward apoptosis. Cytochrome c escapes into the cytoplasm as a result of mitochondrial outer membrane permeabilization (MOMP), where it binds to procaspase-9 and Apaf-1 to form the apoptosome complex [18].

**Figure 1:**
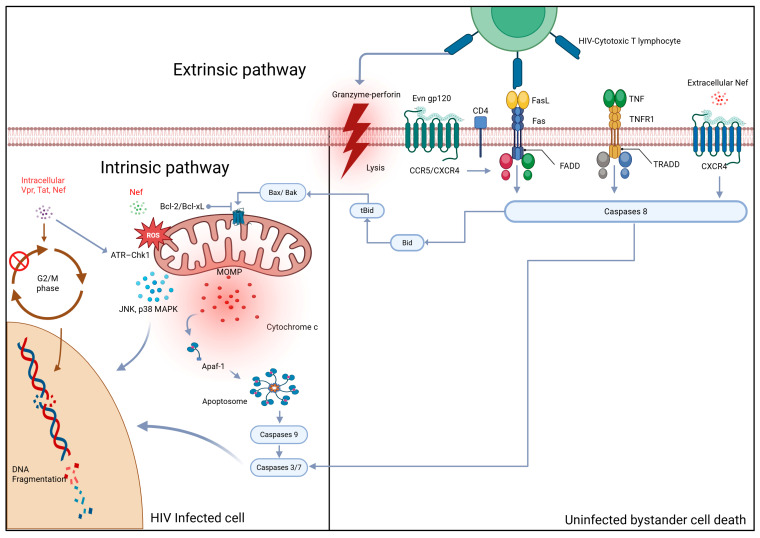
Apoptotic pathways activated in HIV-infected CD4^+^ T helper cells encompass both the extrinsic (death receptor–mediated) and intrinsic (mitochondria-dependent) mechanisms. Key viral proteins driving apoptosis include the Evn gp120 subunit, the Nef, Tat, and Vpr. The Evn gp120 subunit protein binds to the CD4 receptor and engages either the CCR5 or CXCR4 chemokine coreceptors to facilitate viral entry. Beyond entry, this interaction induces FasL expression on the T-cell surface, thereby activating the extrinsic apoptotic pathway. The soluble extracellular Nef protein also interacts with the CXCR4 receptor to amplify apoptotic signaling. Additionally, Evn gp120 subunit interaction with CCR5/CXCR4 upregulates Fas and FasL expression while downregulating anti-apoptotic Bcl-2 family proteins, tipping the balance toward cell death. Simultaneously, the Tat protein reinforces Fas/FasL signaling and activates caspase-8, accelerating apoptotic cascade propagation. Intracellular Tat, Nef, and Vpr disrupt mitochondrial integrity by inhibiting Bcl-2 family members, promoting cytochrome c release, apoptosome assembly, and downstream caspase activation. Moreover, Vpr induces G_2_ cell-cycle arrest, further impairing T-cell survival. Caspases 3/7, together with kinases p38 MAPK and JNK, act on the nucleus, causing DNA fragmentation. CCR5, C–C chemokine receptor type 5; CXCR4, C–X–C chemokine receptor type 4; FasL, Fas ligand; Bcl-2, B-cell lymphoma 2; Vpr, viral protein R; Tat, transactivator of transcription; Nef, negative regulatory factor; MAPK, mitogen-activated protein kinase; JNK, c-Jun N-terminal kinase; G_2_, gap 2 phase of the cell cycle.

The activation of caspase-9 subsequently triggers the executioner caspases (caspase-3, -6, and -7), driving DNA fragmentation, membrane blebbing, and cell dismantling characteristic of apoptosis [25,26]. Additionally, Mitochondrial depolarization also increases ROS production, exacerbating oxidative damage and amplifying death signals [84,85]. As a result, the intrinsic apoptotic pathway thus acts as a convergence point for multiple viral assaults, Env gp120 subunit-mediated receptor signaling, Tat-induced oxidative stress, Nef- and Vpr-driven mitochondrial injury, all resulting in irreversible cell death. The depletion of CD4 T cells and the gradual collapse of immune homeostasis that characterize advanced HIV disease are largely caused by this coordinated activation of mitochondrial apoptosis Figure 1.

**Figure 2:**
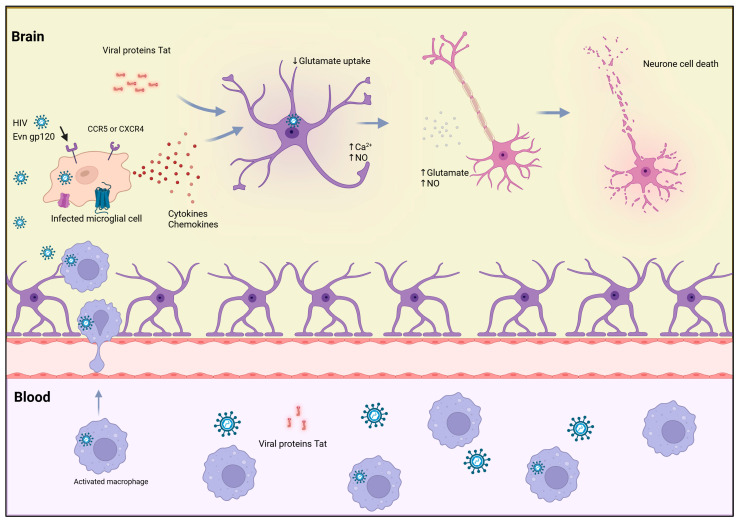
HIV enters the brain via infected macrophages that cross the blood–brain barrier. Once inside, infected macrophages and microglia release viral proteins (Env gp120 subunit), cytokines, and chemokines, which activate uninfected microglia. The activated and HIV-infected cells release neurotoxic substances that cause neuronal injury, dendritic and synaptic damage, and apoptosis. HIV infection also disrupts glutamate regulation, resulting in decreased astrocytic uptake of glutamate, excessive extracellular glutamate accumulation, increased intracellular calcium, and elevated NO production—factors that contribute to excitotoxicity and neuronal death. Both macrophages and neural cells (neurons and astrocytes) express CD4, CCR5, and CXCR4, enabling Evn gp120 subunit-mediated interactions that further drive neurodegeneration. Evn gp120 subunit; CCR5, C–C chemokine receptor type 5; CXCR4, C–X–C chemokine receptor type 4; NO, nitric oxide.

Several studies have shown that infected macrophages are capable of crossing the blood–brain barrier [86] and beyond lymphocytes, Env gp120 subunit-mediated toxicity extends to microglial and neuronal cells, where it induces excitotoxic injury through excessive calcium influx and oxidative stress, linking this mechanism to HIV-associated neurocognitive disorders [87,88,89,90]. This action is triggered by activated macrophages and microglial cells, which secrete viral proteins such as Env gp120 subunit and Tat, along with glutamate and other molecules, including nitric oxide (NO), reactive oxygen species (ROS), cytokines, chemokines, and arachidonic acid, which can directly or indirectly influence glutamate metabolism and transport [90]. Therefore, there is a decrease in glutamate uptake by oligodendrocytes and astrocytes due to increased production of toxins by HIV infected macrophages and microglial cells. Thus, accumulation of excess glutamate induces the activation of glutamate receptors on neurons, leading to elevated intracellular calcium levels, neuronal cell death, and degeneration, Figure 2.

### 2.3. Role of Immune and Inflammatory Mediators

HIV-induced apoptosis is significantly influenced by immune and inflammatory mediators in addition to direct viral effects. HIV infection causes a dysregulated immune response marked by excessive TNF-α, IFN-γ, and IL-1β production, which together create a pro-apoptotic cytokine milieu [91,92]. Long-term exposure to these cytokines can keep lymphoid tissues in a pro-apoptotic state. For example, TNF-α activates caspase-8 to initiate the extrinsic apoptotic pathway when it binds to its receptor TNFR1 on both infected and uninfected cells [43]. Prolonged exposure to IFN-γ increases expression of pro-apoptotic Bcl-2 family members and makes cells more susceptible to Fas-mediated death, ultimately leading to convergence on the mitochondrial intrinsic pathway [93].

This ongoing stress caused by cytokines frequently intensifies into a cytokine storm, a self-amplifying cycle of inflammation and cell death [94]. Even in the absence of productive infection, activated immune cells stimulate bystander CD4^+^ T cells to undergo apoptosis by producing more TNF-α and IFN-γ [30,95]. This mechanism explains why immune-mediated collateral damage drives CD4^+^ T-cell depletion in HIV in addition to direct viral cytopathy.

HIV infection simultaneously disrupts the balance of key survival cytokines. Impaired T-helper cell function reduces interleukin-2 (IL-2), which is necessary for T-cell survival and proliferation, and dysregulates interleukin-7 (IL-7), which is essential for naïve and memory T-cell homeostasis [30]. The PI3K/Akt cascade and other anti-apoptotic pathways are compromised by decreased IL-2 signaling, making T cells more vulnerable to death [96]. The maintenance of resting CD4 T cells is also compromised by altered IL-7 signaling, which leads to lymphoid tissue atrophy and long-term immune depletion. Thus, through chronic cytokine-driven apoptosis, a hallmark of AIDS pathogenesis, the immune systems attempt to suppress HIV infection, paradoxically speeding up immune deterioration.

## 3. Apoptosis as a Host Defense Mechanism

The host immune system employs several biological defense mechanisms against viral infections. Programmed cell death is one of the most immediate and crucial responses. Although apoptosis is a self-sacrificing defense mechanism that eliminates infected cells to stop the virus from spreading, it is pathologically amplified and subverted in HIV infection. However, in infections such as HIV infection, this defense mechanism takes on a paradoxical role. The same pathways designed to protect the host, by apoptosis and its inflammatory counterpart, pyroptosis, are exploited and intensified by the virus, resulting in profound immune system destruction [8,97].

### 3.1. Early Infection Response and Viral Containment

The acute phase of HIV infection serves as an example of how programmed cell death can serve as a containment mechanism at first before ultimately contributing to the immune collapse. Within days to weeks after HIV infection, the gut-associated lymphoid tissue (GALT) dramatically loses CD4 T cells [98,99]. The hosts’ attempt to stop the spread of the virus by sacrificing infected or vulnerable cells is reflected in this early depletion of CD4^+^ T cells, which results from two predominant forms of programmed cell death.

The majority of resting CD4^+^ T cells in lymphoid tissues undergo an abortive form of HIV infection, where reverse transcription starts but is unable to finish [100]. As a result, incomplete viral DNA fragments accumulate and trigger intracellular sensors such as IFI16, which start the formation of inflammasomes and activate caspase-1. Pyroptosis, a very inflammatory type of lytic cell death, is triggered by this cascade series [101]. While pyroptosis can eradicate infected cells, it also releases pro-inflammatory cytokines such as DAMPs and IL-1β, which draw in more immune cells. Due to their susceptibility to infection, these new recruits contribute to the majority of the CD4 T-cell depletion observed during acute infection by sustaining a vicious cycle of inflammation, viral replication, and additional cell death [102]. Therefore, even though pyroptosis evolved to prevent infection, it now plays a significant role in HIV immunopathogenesis.

Caspase-3-dependent apoptosis, a non-inflammatory type of programmed death, kills a smaller percentage of activated CD4 T cells that facilitate full viral replication [100]. This non-inflammatory form of programmed death represents the archetypal “self-sacrifice” biological mechanism aimed at restricting viral dissemination. However, in the broader context of acute HIV infection, apoptosis contributes to only a smaller fraction of the total CD4^+^ T-cell loss when compared to the widespread destruction caused by pyroptosis during acute infection [100].

HIV optimizes its replication and persistence by carefully adjusting host cell death and survival mechanisms. Viral accessory proteins such as Tat, Nef, Vpr, and Vpu have two distinct and context-dependent effects: they can increase the survival of productively infected cells by upregulating anti-apoptotic molecules such as Bcl-2, while also making uninfected bystander cells more susceptible to death [103,104]. Furthermore, the interaction of survival signaling pathways and DNA damage response (DDR) determines the fate of an infected cell. AKT telomerase and ATM/pATM activation patterns determine whether a cell undergoes apoptosis, pyroptosis, or persists to form part of the long-lived viral reservoir [4,105]. As a result, early cell death mechanisms that were initially designed to prevent viral replication are appropriated to increase immunological destruction and inflammation.

### 3.2. Cytotoxic Lymphocyte-Mediated Apoptosis

The Fas/Fas ligand (Fas/FasL) apoptotic pathway and the granzyme–perforin cytolytic pathway are the two main mechanisms by which cytotoxic CD8^+^ T lymphocytes and natural killer (NK) cells eradicate infected targets in the adaptive immune systems antiviral defense against HIV. Nevertheless, widespread bystander apoptosis of uninfected immune cells occurs concurrently with this targeted cytotoxicity during HIV infection. On uninfected T cells, the viral Env gp120 subunit can attach to CD4 and co-receptor molecules (CCR5 or CXCR4), making them more susceptible to Fas-mediated apoptosis [4,8,61]. Furthermore, the expression of death receptors and their ligands is increased by persistent immune activation caused by continuous viral replication and microbial translocation, which is a result of GALT disruption [103,104]. Both infected and uninfected CD4^+^ and CD8^+^ T cells are reduced as a result of this ongoing activation, which sets off activation-induced cell death (AICD). Progressive immunodeficiency is caused by the cumulative loss of CD4 and CD8 T cells, which disturbs immune homeostasis [106,107].

### 3.3. Controlled Apoptosis as an Immunoregulatory Tool

In a successful immune response, apoptosis serves as an immunoregulatory tool to eliminate expanded effector T cells after pathogen clearance, thereby preventing excessive immunity and maintaining homeostasis. This equilibrium is upset when an individual has HIV [108]. The programmed self-sacrifice that typically rebalances the immune system becomes pathological and is exacerbated by prolonged activation. AICD and Fas-mediated apoptosis are enhanced by the long-term immune activation, which modifies cytokine profiles [106,107]. The crucial helper functions for B cells and CD8 T cells are disrupted by the preferential depletion of memory and Th17 CD4 T cells through these death pathways, severely impairing the very immune responses required for viral control and regulation [61]. As a result, the immunoregulatory function of apoptosis is overwhelmed. Instead of restoring homeostasis, it becomes a primary driver of the progressive immune dysfunction and exhaustion that characterizes AIDS, a process that persists even under antiretroviral therapy due to ongoing pyroptosis and inflammation [15]. Thus, apoptosis in HIV infection represents a tragic inversion of the host’s defense, a process meant to protect, turned into a weapon of immune collapse.

## 4. Apoptosis as a Viral Strategy

### 4.1. Bystander Apoptosis and Immune Collapse

**Figure 3:**
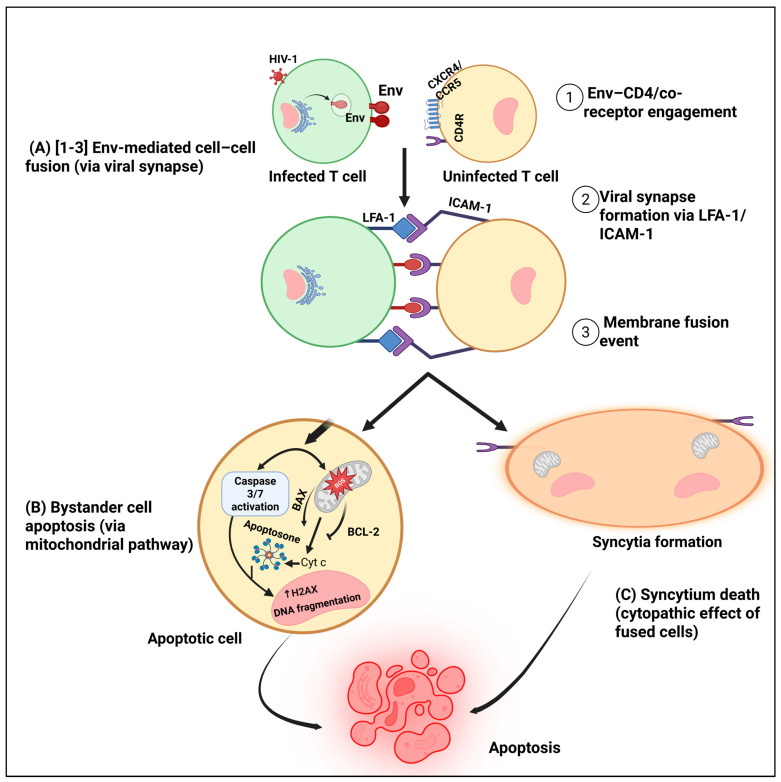
HIV-1 Env-mediated cell-to-cell fusion-initiated apoptosis in uninfected CD4^+^ T cells. HIV-infected T cells expressing Env gp120/gp41 engage uninfected CD4^+^ T cells through three sequential steps: (1) Env binding to CD4, (2) co-receptor (CXCR4/CCR5) engagement and viral synapse formation, and (3) membrane fusion (**A**). This fusion event generates two downstream pathways: (**B**) mitochondrial apoptosis characterized by BAX activation, BCL-2 suppression, cytochrome c release, and caspase-3/7 activation, and (**C**) syncytial instability in fused cells that finally progress to apoptotic death. Both pathways culminate in extensive uninfected CD4^+^ T-cell apoptosis. BCL-2, B-cell lymphoma 2; BAX, Bcl-2-associated X; Cyt c, Cytochrome c.

Bystander apoptosis or the loss of CD4 T cells that are not productively infected is one of the hallmarks of HIV pathogenesis Figure 3 [31]. The viral Env gp120 subunit contributes to this process by engaging CD4, the co-receptor CXCR4, and lymphocyte function-associated antigen-1 (LFA-1) through Intercellular Adhesion Molecule-1 (ICAM-1), triggering Fas/FasL-dependent apoptotic signaling in uninfected cells [109,110]. These interactions activate caspase-3, induce mitochondrial depolarization, and cause DNA fragmentation, leading to the death of otherwise healthy CD4^+^ T cells [15,110]. Notably, uninfected bystander cells rather than productively infected ones account for nearly 95% of CD4^+^ T-cell depletion in HIV infection, underscoring the importance of bystander apoptosis to immune collapse [111].

Further studies have demonstrated that CXCR4 engagement by the HIV-1 Env gp120 subunit promotes intracellular calcium flux and activates the p38 MAPK and JNK signaling cascades, thereby sensitizing CD4^+^ T cells to Fas-mediated apoptosis [112]. This signaling axis contributes substantially to the progressive depletion of CD4^+^ T cells, even when only a small fraction of cells is productively infected. Beyond receptor-mediated signaling, abortive HIV infection in resting CD4^+^ T cells triggers caspase-1–dependent pyroptosis, a highly inflammatory form of programmed cell death that amplifies local immune activation [111]. Recent studies have delineated distinct programmed cell death pathways in productively infected (p24^+^) and bystander (p24^−^) CD4^+^ T cells. In primary human T-cell models, HIV exposure triggers multiple forms of programmed cell death, including apoptosis, pyroptosis, and ferroptosis, each defined by unique molecular signatures in infected and uninfected cells [113]. Bystander cells exhibit greater telomere erosion, increased γH2AX expression, a marker of DNA damage response (DDR) activation, and reduced telomerase activity, corresponding with marked reductions in cell survival [113,114]. Viral entry alone, even in the absence of productive replication, induces DDR signaling and mitochondrial dysfunction, leading directly to apoptotic death in uninfected bystander CD4^+^ T cells [113].

Syncytium formation, which is facilitated by the fusion activity of the HIV-1 Env glycoproteins, is another important mechanism that underlies bystander CD4^+^ T-cell death. The two subunits that make up the HIV-1 Env glycoprotein interact with the CD4 receptor and the coreceptors CCR5 or CXCR4 on target cells. The first subunit is called Env gp120. Membrane fusion is facilitated by the second Evn transmembrane (TM) gp41 [115]. When the Env gp120 subunit interacts with CD4 and the co-receptors CXCR4 or CCR5 on nearby uninfected cells, a conformational shift reveals the Evn gp41 subunit’s fusion peptide, allowing the viral and cellular membranes to merge [116,117]. Env-mediated membrane fusion causes a process called fusion-induced apoptosis or fusoptosis, which is a hybrid of programmed necrosis and apoptosis. Syncytium formation is a physiologically significant process in HIV pathogenesis, not just a cell culture artifact, according to recent in vivo imaging studies. Murooka et al. showed that HIV-1-infected CD4 T cells frequently form multinucleated syncytia and actively interact with nearby lymph node resident cells. Damage-associated molecular patterns (DAMPs) such as ATP and HMGB1 are released by dying syncytial cells. These DAMPs attract and activate additional target cells, starting a vicious cycle of tissue damage and immune activation. Effective cell-to-cell transmission within lymphoid tissues is made possible by these dynamic cell–cell interactions, which enable direct virus transfer through virological synapses. The high motility of infected T cells within lymph nodes further enhances viral dissemination and tissue-wide spread of infection in vivo [118].

Consistent with these findings, mucosal explant studies in both human and macaque models revealed that exposure to HIV-1 or SIV-infected cells results in significantly more efficient viral transmission and productive infection than exposure to equivalent amounts of cell-free virions [119,120]. Therefore, cell-associated viral spread, often involving Env-mediated fusion and syncytium formation, represents a dominant mechanism of viral propagation in host tissues. Such direct cell–cell interactions not only enhance the efficiency of viral transmission but also shield the virus from neutralizing antibodies and innate immune detection, while simultaneously inducing bystander apoptosis in uninfected T cells through fusogenic and signaling-dependent mechanisms [119].

A series of intracellular stress responses, such as mitochondrial depolarization, reactive oxygen species (ROS) accumulation, cytochrome c release, and caspase activation, are involved in this process, also referred to as fusion-induced apoptosis or fusoptosis [121,122]. The mechanical stress of membrane fusion also disrupts cytoskeletal organization and plasma membrane integrity, leading to loss of ion homeostasis and necrotic-like disintegration of the syncytial cell [123]. Additionally, the fusion process amplifies pro-apoptotic signaling, particularly via the Fas/FasL and Bax/Bak pathways, and promotes the activation of executioner caspases-3 and -7, culminating in apoptotic cell death [124].

Recent evidence supports the model that HIV envelope–receptor interactions directly trigger bystander CD4^+^ T-cell death. Inhibition of viral entry, which prevents Env-mediated membrane fusion, restores telomerase activity and AKT signaling while suppressing DNA damage response (DDR) activation in primary CD4^+^ T cells [113]. Conversely, Env-induced membrane fusion provokes mitochondrial dysfunction and DDR activation, leading to caspase-dependent apoptosis in fused cells and inflammatory death in adjacent abortively infected cells [31]. These results show that fusion-related apoptotic and DNA-damage pathways in bystander T cells can be activated by Env–CD4/co-receptor engagement alone, independent of productive viral replication [125]. Thus, gp41-driven membrane fusion causes structural and metabolic collapse, whereas receptor-mediated signaling (gp120–CXCR4/CCR5) sensitizes uninfected cells to apoptosis [31]. When these processes come together, CD4 T-cell loss is accelerated, lymphoid tissue integrity is compromised, and the progressive immune dysfunction associated with chronic HIV infection is exacerbated.

### 4.2. Selective Killing of Immune Regulators

HIV selectively depletes subsets essential for antiviral control while maintaining cells that act as long-lived viral reservoirs by manipulating programmed cell death to undermine protective immune responses. The pool of T cells responsible for coordinating antiviral CD8^+^ and B-cell responses is being eroded by memory CD4^+^ T cells that are specific for HIV antigens, which are disproportionately infected and depleted. These memory cells have higher levels of integrated proviral DNA than other memory subsets and are more vulnerable to activation-induced apoptosis [126].

Interferon-γ-driven antiviral defenses are compromised by the selective depletion of Th1-skewed and central memory CD4^+^ T cells, which encourages viral persistence and immune escape [127]. Macrophages and microglia, on the other hand, are resistant to apoptosis and act as stable viral reservoirs in tissue sanctuaries, particularly in the central nervous system (CNS) [128]. Evidence from in vitro and ex vivo studies indicates that a subset of HIV-infected macrophages withstands acute cytopathic stress by entering a pro-survival state marked by dysregulated apoptotic signaling. These cells show aberrant recruitment or inhibition of the BH3-only protein Bim, yet fail to complete the apoptotic cascade, allowing sustained viral replication and persistence within tissue sanctuaries, particularly in the central nervous system (CNS) [128,129].

HIV enforces this selective survival through active modulation of host death pathways. The viral envelope (Env) upregulates macrophage colony-stimulating factor (M-CSF), which downregulates TRAIL receptor expression (DR4) and induces anti-apoptotic genes such as Bfl-1 and Mcl-1; neutralization of M-CSF or silencing of Bfl-1/Mcl-1 restores TRAIL sensitivity in infected macrophages, demonstrating a concrete mechanism by which Env confers resistance to extrinsic death signals [130]. Furthermore, the accessory protein Nef binds and inhibits the apoptosis signal-regulating kinase ASK-1 (and associated complexes), thereby blunting Fas- and TNF-mediated death signaling in infected cells and promoting survival [131]. Thus, HIV orchestrates a dual strategy, triggering apoptosis in uninfected immune effector cells while suppressing apoptosis in reservoir cells, thereby ensuring both immune collapse and long-term viral persistence.

### 4.3. Hijacking Apoptotic Signaling to Promote Viral Persistence

Beyond selective targeting, HIV modifies apoptotic signaling to promote viral persistence [15]. The virus eliminates immune cells that threaten replication, such as activated CD4^+^ T cells and cytotoxic CD8^+^ T lymphocytes, while preserving long-lived viral reservoirs, including resting memory CD4^+^ T cells and macrophages [61,132]. This dual strategy involves direct modulation of caspases and interference with host survival signaling pathways [70].

A number of viral proteins, such as Tat, Nef, and Vpr, have the ability to directly interact with apoptotic machinery components. For instance, Nef prevents apoptosis by binding and inactivating ASK-1, while Vpr induces mitochondrial depolarization that triggers early apoptotic signals but can also enhance viral gene expression before cell death ensues [15]. The viral Env gp120 subunit activates caspase-8 and -9 cascades in bystander cells, whereas in infected cells, downstream caspase activation is tightly regulated to prevent premature death that would hinder viral replication [125]. On the host side, anti-apoptotic BCL-2 family proteins such as BCL-2, BCL-XL, and MCL-1, which prevent mitochondrial outer membrane permeabilization and caspase activation, are expressed more frequently in HIV-infected cells, especially long-lived memory CD4^+^ T cells [83]. Because of this adaptation, latently infected cells can withstand apoptosis and continue to exist in spite of immune surveillance or antiretroviral therapy. Even though pro-apoptotic Bim is expressed more frequently, its anti-apoptotic counterparts neutralize its function [128]. This imbalance between death initiators and death suppressors is an example of how viruses mimic Bcl-2-like survival control to ensure reservoir longevity.

Additionally, Nef and Tat, two early HIV proteins, directly interfere with the PI3K/Akt signaling pathway to keep infected cells from dying. By attaching to its p85 regulatory subunit, Nef activates PI3K, which phosphorylates Akt and inhibits the pro-apoptotic protein Bad, preserving mitochondrial integrity and cell viability [133,134]. By disrupting PTEN, a negative regulator of Akt, Tat increases this pro-survival effect [135]. Tat inhibits PTEN expression by attaching to p53, a transcriptional activator of PTEN, which results in persistent Akt activation and resistance to apoptosis [135,136]. Tat and Nef work together to create an early anti-apoptotic environment that promotes viral persistence and replication. It has been demonstrated that pharmacologic targeting of the PI3K/Akt pathway, such as Akt inhibition or PTEN function restoration, sensitizes infected lymphocytes to apoptosis while sparing uninfected cells [137].

Beyond infected lymphocytes, HIV-mediated activation of PI3K/Akt signaling occurs in antigen-presenting cells (APCs), where it increases PD-L1 expression and leads to CD8^+^ T-cell exhaustion. Through Akt-dependent transcriptional mechanisms, infected macrophages and dendritic cells exhibit elevated PD-L1 surface expression, which inhibits HIV-specific CD8 T-cell proliferation and cytokine production [96]. By restoring IFN-γ, IL-2, and IL-12 secretion and boosting cytotoxic function, blocking PD-L1/PD-1 interactions reverses this exhaustion phenotype [138,139]. Therefore, through checkpoint upregulation, the same PI3K/Akt axis that inhibits apoptosis in infected cells also indirectly impairs the function of cytotoxic T cells.

On the other hand, chronic activation of the Wnt/β-catenin signaling pathway supports apoptosis resistance in infected lymphocytes. Activation of β-catenin induces the transcription of Bcl-xL, an anti-apoptotic Bcl-2 family protein that stabilizes the mitochondrial membrane and prevents cytochrome c release [140,141]. Experimental induction of β-catenin using the small-molecule agonist 6-bromoindirubin-3′-oxime rescues HIV-infected CD4^+^ and CD4^dim^CD8^bright^ T cells from apoptosis by 40–50%, whereas inhibition of β-catenin with the Wnt/β-catenin pathway reverses this protection. Blocking Bcl-xL using WEHI-539 abrogates β-catenin–mediated survival, underscoring the role of Wnt/β-catenin–Bcl-xL signaling in protecting infected cells from apoptosis [140]. Therefore, both PI3K/Akt and Wnt/β-catenin activation illustrate how HIV reprograms host survival networks to maintain reservoir cells and evade immune clearance.

## 5. Chronic Immune Activation and Pyroptosis

### 5.1. From Apoptosis to Pyroptosis

Our understanding of CD4^+^ T-cell depletion in HIV infection has evolved significantly, recognizing that pyroptosis rather than classical apoptosis predominates in chronic infection [142,143]. Pyroptosis is a type of inflammatory programmed cell death that helps the body respond to infection or cellular stress. It is triggered when inflammasomes, large protein complexes that sense danger signals inside the cell, activate caspase-1. The inflammasome-activated caspase-1 triggers it by cutting gasdermin family proteins into membrane holes. This results in swelling, lysis, and release of pro-inflammatory cytokines IL-1β and IL-18 [143,144]. This inflammasome-dominated disease contrasts sharply with apoptosis, which is usually immunologically silent. Caspase-3 activation without membrane disruption normally precipitates a flow of chemical events, death-inducing for the individual cell, but unlikely to affect neighbors.

Recent studies of CD4^+^ T cells apoptosis in patients with HIV infection have uncovered two major populations of cells, the pyroptotic type (positive for caspase 1) and the apoptotic type (positive for caspase 3), with different looks under the microscope and different responses to ARVs [142]. Flow cytometry analyses on Lymphoid tissue have revealed that active gasdermin-D staining for pyroptosis could be demonstrated directly in lymph node tissue sections, supporting the conclusion that this kind of cell death process does happen in vivo [142]. Importantly, untreated infection throughout its course is distinguished by pyroptosis predominating in both side bystander and cell death, following successful antiretroviral therapy, this situation changes. Now also includes serving as an indicator for inflamed comorbidities [142,145].

Flow cytometry on lymphoid tissue has shown that active gasdermin-D staining to mark pyroptosis can be identified directly within lymph node sections. This evidence is consistent with the conclusion that there is pyroptotic cell death within the lymphoid tissues in vivo [142,143].

### 5.2. Role of Innate Immune Sensing and Inflammasomes

Pyroptosis is induced by HIV infection by the perception of viral ligands and DAMPs by the innate immune. This causes inflammasome assembly and the activation of caspase-1. In the latest publications, several inflammasome sensors are involved with HIV-induced inflammasome activation, and NLRP3 and CARD8 are leading the charge [142,146].

Patient-centered studies have uncovered extensive NLRP3-dependent caspase-1 activation within circulating CD4^+^ T cells and lymph node cells. Such activation is closely related to chronic HIV infection, where priming signals such as ROS generation drive the assembly of the NLRP3 inflammasome complex. This process eventually leads to the activation of caspase-1, an integral event leading to the initiation of an inflammatory programmed cell death called pyroptosis [142,144]. NLRP3 inflammasome appears particularly sensitive to the cell stress and mitochondrial damage that are characteristic of HIV infection and therefore create an ideal setting supportive of immune cell apoptosis. Of further interest is the recent mechanistic finding wherein the CARD8 inflammasome is potentially directly activated through the action of HIV-1 protease [146]. This discovery shows that there is a molecular link between the viral replication of HIV and the activation of the inflammasome, and illustrates the way that HIV uses the innate immunity sensors of the cell to trigger inflammation and ensure its survival. CARD8 is an intrinsic sensor entity capable of monitoring viral protease action and thereby inducing pyroptosis and inflammatory response. This discovery is an important insight into how HIV directly manipulates and disrupts the death pathways of the host cell and thus influences the dysregulation within the immune system and onward disease progression.

Single-cell tissue analysis has also shown that subsets of CD4^+^ T cells that are susceptible to abortive infection have higher levels of transcripts for inflammatory caspases and gasdermin-D, positioning them as highly sensitive bystander targets of inflammasome activation and pyroptosis within lymphoid tissues [147]. This selective vulnerability explains the loss of particular CD4^+^ T-cell subsets in HIV infection.

Inflammasome activation has downstream consequences in addition to cell death. In HIV-related chronic inflammation, bioactive cytokines that are produced when caspase 1 processes the zymogens pro-IL-1β and pro-IL-18 are essential. Strong associations between caspase-1 activation and increased inflammatory markers, such as increased levels of IL-β and IL-18 in the plasma of HIV-positive individuals, have been found in clinical studies [142].

### 5.3. The Vicious Cycle: Immune Activation, Death, and Tissue Damage

The persistent activation of inflammasomes, particularly NLRP3 and CARD8, has also been posited to trigger an autonomous pathogenic loop characterized by sustained immune activation, mucosal destruction, and microbial translocation. Such a harmful cycle is among the greatest hurdles to HIV pathogenesis since it can persist despite the effective control of viruses by antiretroviral therapy (ART) alone. Ongoing inflammatory microenvironment sustained by pyroptosis signaling widens progressive tissue damage and immune dysregulation, and therefore accentuates disease progression and the development of non-AIDS comorbidities [142,148].

Mechanistically, this cycle operates through various interlinked pathways. Activation of caspase-1 within inflammasomes causes the cleavage of gasdermin-D, leading to the generation of membrane pores, inducing cell swelling and lysis and the production of strong pro-inflammatory cytokines, namely interleukin-1β (IL-1β) and interleukin-18 (IL-18). Elevated plasma concentrations of these cytokines demonstrate a strong correlation with the systemic inflammatory markers among individuals diagnosed with HIV [142]. Egress of the IL-1β and IL-18 increases the recruitment and activation of immune cells and sustains an inflammatory state that further destroys mucosal integrity, especially within the gastrointestinal tract.

The disruption of the epithelial barrier, increased intestinal permeability, and dysbiotic changes in gut microbiota have all been closely linked to prolonged immune activation. These changes facilitate the translocation of microorganism components such as lipopolysaccharide (LPS) into the bloodstream, thereby sustaining the inflammatory process and immune response activation. This supporting evidences show elevated serum levels of gut damage biomarkers (such as intestinal fatty acid-binding protein) and significant differences in fecal microbiome and metabolome profiles in HIV-positive people [148,149,150].

This impaired gut barrier function is an improper translocation of microorganisms, leading to a rise in markers reflecting bacterial or yeasts (lipopolysaccharide[LPS], soluble CD14[scd14], and β-D-glucan[bglu]). Such disruption of gut barrier function has been linked to chronic systemic immune activation amongst people infected with HIV [148,150]. Ongoing transfer of intestinal microbial products then provides additional stimulants for tissue immune cell exposure and inflammasome activation, driving caspase-1-dependent cell death and maintaining a self-sustaining pathogenic cycle [113,142].

Clinical studies have also suggested relationships between dysbiotic bacteria and microbial metabolites and the parameters of microbial translocation and immune activation across different stages of HIV disease progression [149,150]. Significantly, interventions to specific aspects of this cycle also represent the potential to break the pathogenic cycle of feedback. Well-tolerated antiretroviral therapies, involving regimens containing integrase inhibitors, together with microbiome-directed strategies, themselves have shown the potential to partially restore indicators of microbial translocation (such as TLRs and nucleosomes), inflammation (such as IL-6 and hsCRP), and gut permeability within clinical cohorts. This evidence suggests that interventions to the upstream determinants of this cycle can prevent its progression [151,152].

## 6. Therapeutic Implications

### 6.1. Effects of Antiretroviral Therapy (ART) on Apoptotic Pathways

In HIV-positive people, ART affects apoptotic pathways, especially through effects on immune cell activity and mitochondrial function. ARTs therapeutic effects and, in certain situations, its harmful toxicities are linked to changes in the expression of genes related to apoptosis [153]. By blocking mitochondrial DNA polymerase gamma, nucleoside reverse transcriptase inhibitors (NRTIs) can cause mitochondrial dysfunction and toxicity [153]. Twenty-six apoptosis-related genes are expressed differently in HIV-positive patients with ART-associated mitochondrial toxicity compared to uninfected controls according to case–control studies. Among these, DFFA and TNFRSF1A were markedly elevated and accurately identified 75–86% of subjects as cases or controls, indicating their potential as biomarkers [153,154]. A shift in apoptotic signaling networks was indicated by network analysis, which showed that affected individuals had changed gene interaction patterns with Fas Ligand (FASLG) playing a key role [154]. ART partially restores immune function in HIV-positive individuals by modulating immune cell apoptosis [154].

ART modulates apoptosis in immune cells, contributing to the partial restoration of immune function in people living with HIV [155]. HIV infection increases programmed cell death in lymphocytes and neutrophils, thereby impairing immune responses such as neutrophil antimycobacterial activity. Despite this, ART reduces excessive apoptosis, promoting more physiological cell death patterns and helping control viral replication [155,156]. However, incomplete immune recovery persists in 10–40% of ART-treated individuals, referred to as immunological non-responders (INR), due in part to continued extrinsic pathway-mediated apoptosis involving Fas/FasL and caspase-3 [157]. Studies also demonstrate there is a significantly increased expression of the genes involved in apoptosis in the INR, such as CASP3, FASLG, in comparison to the immunological responders, which accentuates the importance of the destruction of apoptotic cells in regulating the restoration of the CD4^+^ T cells [158]. Chronic immune activation, as well as the associated chronic inflammation in individuals taking ART, is also fueled by mitochondrial dysfunction, which stimulates the secretion of reactive oxygen species and oxidized mitochondrial DNA, thereby reducing the inflammatory response [159]. Moreover, the TLR7-IRF5 axis increases the susceptibility of the memory CD4^+^ T cells in individuals infected with the Fas apoptosis [160].

The modulation of cellular pathways of programmed cell death has proved to be a promising approach in the conservation of the immune function in HIV/AIDS, as it prevents the depletion of CD4^+^ T lymphocytes as well as the chronic inflammatory process associated. Caspase inhibitors, particularly those targeting caspase-1 and caspase-8, have demonstrated potential in reducing pyroptosis and apoptosis in HIV-infected cells. A recent study has shown that the caspase-1 inhibitor VX-765 effectively mitigates pyroptosis and apoptosis, thereby decreasing CD4^+^ T-cell depletion and associated inflammation. This study evaluated the effects of VX-765 in HIV-1–infected humanized NSG mice engrafted with human CD34^+^ hematopoietic stem cells and found that treatment significantly reduced the expression of pro-inflammatory cytokines, including TNF-α and IL-18, preserved CD4^+^ T-cell populations, and decreased both viral load and total HIV-1 DNA in the spleen. Elevated caspase-1 expression has been associated with poor immune recovery in people living with HIV, suggesting that selective caspase inhibition may help preserve immune function. Nevertheless, broad caspase inhibition carries potential risks, including disruption of normal immune responses and physiological cell turnover.

### 6.2. Targeting Apoptosis and Pyroptosis for Immune Preservation

IL-1β, a pro-inflammatory cytokine released during pyroptosis, plays a central role 770 in driving immune activation and tissue damage in HIV-infected individuals [161]. Various studies demonstrated the activation of the inflammasome in HIV-infected individuals, where IL-1β expression was significantly up-regulated in monocytes and macrophages [162,163]. This chronic inflammatory process has been implicated in sustained immune activation, endothelial dysfunction, and injury, even in individuals undergoing antiretroviral therapy. IL-1β-targeted interventions in animal models, using pharmacological inhibition, have been demonstrated to decrease systemic levels of inflammation as well as restore immune homeostasis. A pilot clinical trial indicated that IL-1β inhibition in HIV-infected individuals reduced inflammatory parameters and improved endothelial function, while mechanistic studies revealed IL-1β blocked signaling as effective in reducing inflammasome-mediated activation of CD4^+^ lymphocyte loss and injury [164]. Although there has been evidence suggesting the lack of significant variation in IL-1β levels, the IL-1 family of cytokine-targeted interventions may help in reducing immune-mediated injury [165]. Clinical trials assessing IL-1β blockers in HIV-infected individuals are in progress [166].

Apoptosis and pyroptosis, as targets, also have potential benefits as well as limitations. The inhibition of these mechanisms could help maintain the level of CD4^+^ T cells, promote immune reconstitution, as well as decrease chronic inflammatory conditions, thus slowing the progression of the disease, as well as the incidence of comorbidities [69]. On the other hand, the inhibition of apoptosis could result in the accumulation of infected and/or damaged cells, as well as the impairment of the immune system, in addition to rendering the individual susceptible to opportunistic infections/malignancies [69,161]. Therefore, careful modulation is essential to balance immune preservation with necessary physiological cell clearance.

### 6.3. Implications for HIV Cure Strategies

Apoptosis is integral to HIV eradication strategies, as it mediates the relationship between the elimination of infected cells and the conservation of the immune response. In the shock-and-kill strategy, HIV latent infection is exploited using latency-reversing agents (LRAs) that make infected cells sensitive to the immune response or the cytopathic effects of the virus [167,168]. Nonetheless, the strategy can neglect the potency of apoptosis, as the use of some LRAs can activate survival signaling [167]. Moreover, HIV takes advantage of the anti-apoptotic protein BCL-2 to create latent viral reservoirs [168]. The use of LRAs in combination with pro-apoptotic agents, BCL-2 inhibitors such as venetoclax, can make infected cells sensitive to apoptosis while leaving the immune response unaffected [169].

The block-and-lock strategy, on the other hand, targets a functional cure based on the irreversible inhibition of HIV transcription, reactivation, cytopathic, and immune activation. Apoptosis is also reduced to a minimum, thereby preserving the immune cells, although it has not been able to target the latent HIV reservoirs. Didehydro-Cortistatin A (dCA) compounds, a Tat inhibitor, can decrease HIV-1 transcription as well as induce deep latency without causing apoptosis in non-infected cells [170].

The difficulty in these strategies exists in the selective induction of apoptosis in infected cells, rather than harming the non-infected immune cells [167]. The survival mechanisms in HIV-infected cells include BCL-2, which can be leveraged to promote the selective apoptosis of infected cells [168]. The usage of a combination therapy regime comprising LRAs, apoptosis-modulating agents, as well as immune potentiators has been explored in the effort to strike the right balance between the two [171]. Studies conducted using BH3 mimetics, which targeted BCL-2 protein family members, showed increased apoptosis in HIV-infected cells without harming the uninfected cells, which could be a strategy to make the shock-and-kill approach more effective [168].

## 7. Conclusions

A paradox at the core of viral pathogenesis is embodied by HIV-induced apoptosis: a host defense mechanism that the virus takes advantage of to compromise immune integrity. Apoptosis and pyroptosis function as innate containment responses in the early phases of infection intended to eradicate infected cells and limit the spread of the virus. But HIV deftly manipulates these same pathways, turning programmed cell death into a catalyst for immune collapse. The virus amplifies CD4 T-cell depletion by orchestrating both direct cytopathic injury and bystander apoptosis through viral proteins such as Tat, Nef, Vpr, and Env gp120 subunit. Even with successful antiretroviral therapy, the persistence of pyroptosis and inflammasome activation impedes immune reconstitution and prolongs chronic inflammation.

Apoptosis’s dual role as a protector and a destroyer highlights how intricately HIV interacts with host defenses. Effective treatment approaches should target inflammatory and cell death pathways in addition to viral suppression. By focusing on dysregulated apoptosis and pyroptosis, it is possible to improve cure tactics such as shock-and-kill and block-and-lock, limit reservoir formation, and maintain immune function. In order to achieve long-lasting immune restoration and possibly functional eradication of HIV, it is crucial to reconcile the opposing roles of apoptosis in HIV infection, balancing immune protection with viral persistence.

## 8. Future Directions

A profound paradox in viral pathogenesis is presented by the intricate interaction between HIV and host cell death pathways. HIV systematically manipulates apoptosis, a basic defense mechanism intended to eradicate infected cells and stop the spread of the virus to cause immune collapse. Even though there has been a lot of progress in understanding these pathways, integrated therapeutic-focused studies must replace observational studies in the future. The isolated pathways involved in viral pathogenesis have been the main focus of recent research. Beyond these, a more comprehensive multi-omics strategy that aims to combine various methods such as transcriptomics, proteomics, epigenomics, and metabolomics should be used in the future. These can be used on distinct cellular subsets from various tissue reservoirs, such as latently infected, productively infected, and bystander cells. The mapping of dynamic regulatory networks that determine cell fate, such as survival, apoptosis, and pyroptosis, will be made easier by such thorough analyses.

Furthermore, understanding how host signaling networks, such as the Bcl-2 family, balance DNA damage response pathways and inflammasome activation, interact with viral proteins such as Tat, Nef, and Vpr may uncover new and valuable therapeutic targets for intervention. Even so, a large portion of our current knowledge comes from peripheral blood analyses or in vitro models, which fall short of capturing the intricacy of tissue-specific interactions. Advanced technologies such as spatial transcriptomics on human lymphoid tissue biopsies and multiphoton intravital microscopy in humanized mouse models should be used in future studies. This method makes it possible to see and examine dynamic biological processes in living things’ tissues at the cellular and subcellular levels. Within the architectural framework of lymph nodes and GALT, the main locations of CD4 T-cell depletion during HIV infection include syncytia formation, virological synapse activity, and pyroptotic cell death.

Although broad viral immunosuppression has been the main focus of therapeutic strategies up to this point, precision interventions that specifically modify cell death pathways should be given priority in future approaches. The goal should be to create agents that can perform two complementary actions. First, selectively protecting bystander cells by blocking pyroptosis either by enhancing anti-apoptotic signaling in uninfected cells or by targeting important mediators such as caspase-1, NLRP3, or gasdermin-D. Second, employing priming agents such as BH3 mimetics to selectively eradicate reservoir cells. These agents lower the apoptotic threshold in latently infected cells, making them more susceptible to immune clearance or latency-reversing agents in shock-and-kill strategies. These focused treatments may eventually break the cycle of inflammation and bystander cell death that continues in spite of ART, maintaining immune function without unintentionally protecting the viral reservoir. The best windows for therapeutic intervention should be determined by future studies. Preclinical data suggests that the best way to prevent reservoir establishment and maintain CD4^+^ T-cell populations may be to administer cell death modulators early either during acute infection or at the start of ART.

Furthermore, systematic testing in reliable animal models is necessary when combining these modulators with complementary approaches such as immune checkpoint inhibitors, therapeutic vaccines, or latency-reversing drugs. In order to establish synergistic regimens that improve viral clearance while reducing toxicity and maintaining immune homeostasis, such studies are crucial. The need for trustworthy predictive biomarkers is underscored by the varied responses to ART. Future research should concentrate on confirming markers of mitochondrial stress and apoptotic priming in addition to indicators of inflammasome activation, such as caspase-1 activity and IL-18 levels. By establishing such biomarkers, patients who are most likely to benefit from adjunctive therapies that target cell death pathways can be stratified, especially immunological non-responders.

With the help of this precision-based approach HIV management may progress toward more individualized and successful treatment approaches. By pursuing these lines of inquiry, the field will be able to convert a comprehensive mechanistic understanding of HIV-induced cell death into revolutionary therapeutic approaches that accomplish both long-term viral control and full immune reconstitution.

## Data Availability

Not applicable.
